# Fungal infections lead to shifts in thermal tolerance and voluntary exposure to extreme temperatures in both prey and predator insects

**DOI:** 10.1038/s41598-021-00248-z

**Published:** 2021-11-05

**Authors:** Mitzy F. Porras, Gustavo A. Agudelo-Cantero, M. Geovanni Santiago-Martínez, Carlos A. Navas, Volker Loeschcke, Jesper Givskov Sørensen, Edwin G. Rajotte

**Affiliations:** 1 Department of Entomology, The Pennsylvania State University, 501 ASI Bldg., University Park, PA 16802 USA; 2 Department of Physiology, Institute of Biosciences, University of São Paulo, Rua do Matão 101, Tv 14, São Paulo, 05508-090 Brazil; 3 Department of Biology – Genetics, Ecology and Evolution, Aarhus University, Ny Munkegade 116, 8000 Aarhus C, Denmark; 4 Department of Biochemistry, The Pennsylvania State University, 308B Althouse Lab., University Park, PA 16802 USA

**Keywords:** Animal behaviour, Animal physiology, Entomology, Ecology, Ecophysiology

## Abstract

Pathogens can modify many aspects of host behavior or physiology with cascading impacts across trophic levels in terrestrial food webs. These changes include thermal tolerance of hosts, however the effects of fungal infections on thermal tolerances and behavioral responses to extreme temperatures (ET) across trophic levels have rarely been studied. We examined how a fungal pathogen, *Beauveria bassiana*, affects upper and lower thermal tolerance, and behavior of an herbivorous insect, *Acyrthosiphon pisum*, and its predator beetle, *Hippodamia convergens*. We compared changes in thermal tolerance limits (CT_Min_ and CT_Max_), thermal boldness (voluntary exposure to ET), energetic cost (ATP) posed by each response (thermal tolerance and boldness) between healthy insects and insects infected with two fungal loads. Fungal infection reduced CT_Max_ of both aphids and beetles, as well as CT_Min_ of beetles. Fungal infection modified the tendency, or boldness, of aphids and predator beetles to cross either warm or cold ET zones (ETZ). ATP levels increased with pathogen infection in both insect species, and the highest ATP levels were found in individuals that crossed cold ETZ. Fungal infection narrowed the thermal tolerance range and inhibited thermal boldness behaviors to cross ET. As environmental temperatures rise, response to thermal stress will be asymmetric among members of a food web at different trophic levels, which may have implications for predator–prey interactions, food web structures, and species distributions.

## Introduction

Fungal pathogens and temperature can constrain the physiology and behavior of insects, with implications for ecological interactions, such as predation, that can ultimately affect populations across trophic levels and food webs^1,2^. Several insect species regulate body temperature both physiologically and behaviorally by selecting environmental temperatures. In turn, behavioral thermoregulation may be affected by interspecific relationships, including those of a pathogenic nature. For instance, host exposure to temperatures near thermal limits may alter foraging behavior, survival, and reproduction^3–5^, but also host–pathogen interactions. Pathogens may alter host thermoregulationby eliciting changes in behavior and physiology^6^. For example, behavioral fever (voluntary exposure to warmer than normal body temperature) is seen as part of the immune response of insects, at least in some species^7,8^. In parallel, pathogen-induced exposure to temperatures bordering thermal limits (extreme temperatures, ET hereafter) may reduce insect immune response, thus increasing infection susceptibility^9^. Under this framework, the effects of ET on insects and their pathogens seem poorly understood, particularly regarding the impact of fungal pathogens on thermal behavior^10^.

Predator beetles and aphids’ responses are shaped by abiotic factors, ecological interactions with other species such as pathogens, and navigation through ET within thermal landscapes. Pathogens and exposure to ET can influence behavior and life cycles of both prey and predator, as well as the dynamics of fungal infections on both species^11^. However, ET is relative to species-specific thermal physiology; therefore, differences in ET tolerance are expected between host and pathogens. For example, the lower and upper-thermal limits of *Beauveria bassiana* are 8 °C and 33 °C, respectively^12^, with an optimal range between 25 and 28 °C^13^, whereas adults of *Acyrtociphon pisum* have a minimum thermal limit of 10 °C and a minimum limit of 40 °C^14^. The counterparts for the predator beetle, *Hippodamia convergens*, are 6.5 °C and 50 °C, respectively^15,16^.

The incidence of suboptimal temperatures and extreme thermal events may alter organismal function leading to changes in trophic cascades^17^. For example, exposure to ET reduced foraging, depressed rates of attack, and decreased prey consumption in rotifers, dragonfly larvae, and fishes^18–20^. Extreme thermal events can alter survival and virulence of fungal pathogens^21^ and other aspects of pathogen-insect interactions^6^. For example, insects infected by pathogens (e.g., *Metarhizium anisopliae or Rikettsiella grylli*) select temperatures that reduce pathogen virulence (behavioral fever)^6,8^. Pathogen-infected insects can induce behavioral fever by selecting temperatures found throughout the thermal landscape, however, selecting temperatures higher than the maximum limit pose acute constraints to organismal function^22^.

Facing suboptimal thermal variation may affect cell homeostasis by reducing oxygen supply and altering membrane fluidity, leading to increased oxidative stress. Such changes may alter the energy supply and ATP synthesis^23^. Although the energetic cost of insects’ thermal tolerance has been well studied, our understanding of the impact of multiple stressors (fungal infection and thermal variation) is only partially understood.

This work examined whether fungal infections in insects affect thermal tolerances and behavior. We hypothesized that a fungal infection reduces thermal tolerance range and induces shifts in voluntary exposure to ET (thermal boldness) in both predator and prey insect species^24^. We predict that fungal infection reduces the maximum thermal tolerance (CT_Max_), increases the low counterpart (CT_Min_) and reduces voluntary exposure to warm ET. We expect fungal infection to alter the predator–prey system through differential impacts on each species’ thermal behavior and the energetic cost associated with exposure to critical thermal limits. To test this hypothesis, we chose a predator–prey system composed of the predator *H. convergens,* its prey *A. pisum*, and a fungal pathogen, *B. bassiana*, which can infect both insect species. We experimentally infected aphids and predator beetles with the fungal pathogen using spore solutions at different concentrations applied in the field. We then collected individuals and assessed thermal tolerances (CT_Max_ and CT_Min_) of healthy and infected aphids and predator beetles under controlled conditions in the laboratory. Next, we examined whether infection altered thermal boldness using species-specific extreme temperatures in order to characterize the impacts of infection on thermal boldness (voluntary crossings of ET). Finally, we determined energetic costs (ATP levels) associated with each physiological and behavioral response in healthy and infected insects as an indicator of the energetic cost of reaching or exploring critical thermal limits.

## Material and methods

### Field trials

Field trials were conducted in three raised beds (1 × 2 × 0.6 m) on the Penn State University campus from July to August 2020. The raised beds were separated by at least 8 m to avoid treatment cross-contamination. Faba bean (*Vicia faba* L.) seeds were planted at a density of 20 seeds/ m2 (50 plants per bed), and each bed was caged using a metal-framed tent. “Noseeum” nylon mesh (Outdoor Wilderness Fabric s, Inc., Caldwell, ID) was draped over the frame and the edges buried in the soil of the bed. The sides of the cages were fastened closed with zippers to allow access.

### Insects

Aphid and predator beetle colonies were raised separately on faba bean plants in cages (BugDorm 20 cm × 40 cm × 20 cm, BioQuip Products, Inc., Rancho Dominguez, CA) in the field. Larvae and adults of predator beetles were fed with a combination of *A. pisum* and *Rhopalosiphum padi* every other day (Supplementary information Fig. S1). Trials involving plants, insects, and entomopathogenic fungi were conducted according to institutional, national, and international guidelines and legislation.

### Fungal inoculations (*Beavueria bassiana*)

We released first instar aphid nymphs on each faba bean plant on the raised beds (~ 1100 aphids) by gently shaking plastic containers with groups of 20 nymphs and placing them on the plants using a paintbrush. They were allowed to grow and reproduce for fifteen days. During the night, we sprayed spore suspension of the *Beauveria* strain GHA (BotaniGard ®, MT, USA) at 1.4 × 10^6^ and 1.4 × 10^12^ spore ha^−1^, low and high load respectively. Two days after inoculation, we collected adult aphids (~ 4–5 days old) from the experimental plots and measured physiological parameters (see details below). Next, we released 300 adult beetles inside each aphid–fungal inoculated cage, allowed them to feed for 2–3 days in our experimental cages, and then collected beetles for physiological measurement.

### Identification of critical thermal limits (CT_Max_ and CT_Min_) of healthy and infected insects

To determine critical thermal maximum for locomotion (CT_Max_) of healthy and infected individuals of each species, we employed a protocol modified from Ribeiro et al.^25^, using a hotplate with a programmable heating rate controlled by a computer interface (Sable Systems, LV, USA). The temperature was monitored by independent thermocouple channels connected to a Hobo 4-channel data logger. One thermocouple was attached to the surface of the hotplate, and the other sensor was attached inside the glass tube plugged by a cotton ball in which we placed an individual insect. This equipment was located inside an automated thermal chamber (interior dimensions: width 40.5 cm × 35 cm length × 40 cm height). We transferred an adult aphid (4-day-old) into the glass tube and exposed it to increasing temperatures at a rate of 0.3 °C min^−1^ until its locomotion stopped. CT_Max_ was recorded when the insect turned upside down and could no longer return to the upright position within 5 s. The insect was returned to a faba bean leaf for recovery (*n* = 10 individuals per treatment).

To measure the critical thermal minimum for locomotion (CT_Min_) of healthy and infected individuals of each species (*n* = 10 individuals per treatment), we used an insulated incubator where the temperature was monitored by independent thermocouple channels connected to a Hobo 4-channel data logger. The sensors were attached inside three glass tubes, each tube with an adult (3 to 4-day-old), and plugged by a cotton ball. The glass tube was exposed to decreasing temperature at a rate of 0.3 °C min^−1^ until its locomotion stopped. CT_Min_ was recorded when no movement was recorded within 5 s. The insect was returned to an aphid-infested faba bean leaf for recovery. Data were only considered valid if the insect displayed normal activity 2 h after a CT_Max_ or CT_Min_ test.

### Impacts of infection on voluntary exposure aphids and predator beetles to extreme thermal zones

To examine how voluntary exposure to ETZ was affected by fungal infection, we collected aphids and predator beetles (3 to 5 day-old) from our field plots and transferred them to a dark plastic bottle. Next, a bottle containing the insects was attached to a choice test arena following a modified protocol from Navas et al.^24^. This experimental arena allows insects to freely move across extreme temperatures to access food in containers located at each end of the device. To reach food, individuals had to cross an ETZ, either warm or cold. The location of each insect was recorded after 60 min, and it was classified as: exploration for individuals that left the initial black bottle, warm or cold ETZ crossings. The experiment was replicated ten times for each species and treatment condition [aphid: healthy, infected (low and high spore load); predator beetle: healthy, infected (low and high spore load)].

### Effects of fungal infection and thermal conditions (critical thermal limits and voluntary exposure to ETZs) on longevity of aphids and predator beetles

To examine whether fungal infection and thermal conditions alter longevity in aphids and beetles, we isolated three individuals from each factor combination (low, high fungal load, CT_Min_, CT_Max_, behavior: crosses to ETZ cold, warm, and no cross) from previous experiments, and counted the number of days the adults survived after the exposure to the thermal condition (*n* = 3 factor combination).

### Energetic cost associated with fungal infection of aphid and predator beetles under critical thermal limits and voluntary exposure to ET

Intracellular ATP content was determined in neutralized perchloric acid extracts and by a spectrophotometric coupled enzyme assay, based on modified protocol from Churchill and Storey^26^ content (*n* = 3 per treatment condition). An insect was ground to powder using a mortar and pestle cooled in liquid nitrogen, and then weighed into 1.5 mL microcentrifuge tubes (Eppendorf). Powder was dissolved with 0.1 mL ice-cold TE buffer (50 mM Tris–HCl, pH 7.5 plus 1 mM EGTA) and homogenized by sonication (15 s, three times), using a Q500 Sonicator system (QSonica, Newtown, CT, USA). An aliquot (10 µL) of the well-mixed homogenate was removed for protein determination. Cells were lysed by adding 6% (v/v) ice-cold perchloric acid, strongly vortexed for 2 min and incubated at 4 °C for 10 min. Next, the cell homogenate was centrifuged at 14,462 rpm and 4 °C for 5 min. The resulting supernatant was neutralized by adding KOH/Tris (3 M/0.1 M) and centrifuged again to discard the perchlorate salts. Extracts were kept at 4 °C for their immediate utilization. ATP content was determined spectrophotometrically by following the production of NADPH at 340 nm (ε = 6.22 mM^−1^ cm^−1^) and using CARY WinUV-Vis Spectrophotometer (Agilent, Santa Clara, CA, USA). The following reagents were used for the spectrophotometric coupled enzyme assay: 5 U Hexokinase, 10 U Glucose 6-phosphate dehydrogenase, 1 mM NADP + , 5 mM MgCl2 and 10 mM Glucose in HE buffer (100 mM Hepes-HCl plus 1 mM EGTA, pH 7.0) at 25 °C. Chemicals were purchased from Roche (Manheim, Germany) and Sigma (St Louis, MO, USA).

### Infection status

We used two different protocols to confirm fungal infection: (1) placing each individual in wet towel paper inside a Ziploc bag to observe hyphal growth^27^. (2) For insects used in ATP measurements, we followed a modified protocol from Wraight and Ramos^28^ and Castrillo et al.^29^. Insect were washed using a serial dilution technique, vortexed for 10 s, and mounted in a drop of lactophenol blue, diluted with distilled water. We then preserved insect body parts (i.e., legs and abdomen terga) at − 80 °C for 12 months and placed in Petri dishes containing potato dextrose agar (PDA HiMedia-GM096) medium (pH 6.8), and incubated for ten days. To confirm infection by *B. bassiana*, we observed plates every 3 days, identified fungal growth (dense white mycelia), then randomly chose three samples, collected mycelia, and DNA was extracted using PureLink Genomic DNA Kit (Invitrogen by Thermo Fisher Scientific, Waltham, MA, USA), according to manufacturer’s protocol. Next, we used PCR essays (25 µL) contained 1 × Q5 Hot Start High-Fidelity Master Mix (New England BioLabs), following a protocol modified from Castrillo et al.^29^ using primers GHTqF1 (5′-TTTTCATCGAAAGGTTGTTTCTCG) and GHTq R1 (5′-CTGTGCTGGGTACTGACGTG) amplified a 96-bp region of the SCAR fragment. The PCR protocol was initial denaturation at 98 °C, followed by 30 cycles at 98 °C for 1 min, annealing at 58 °C for 1 min; and extension at 72 °C for 1 min. PCR products were visualized in a 1.0% (wt/vol) agarose gel stained with ethidium bromide.

### Data analysis

All data were tested for statistical test assumptions using a qqplot, Levene's homogeneity test and the Shapiro–Wilk normality test at alpha = 0.05 significance level. For critical thermal limits (CT_Max_ and CT_Min_) experiments, the data sets were non-normal and transformation did not normalize the residuals, so we used nonparametric ANOVAs (Kruskal–Wallis) followed by post-hoc nonparametric multiple comparisons. For voluntary exposure to ETZs, we used a generalized linear model with treatment (healthy, low and high spore load) with Poisson distribution, followed by comparisons within each treatment group. For healthy insects, we used a *t*-test to compare crosses between warm or cold ETZs; for infected insects, we conducted ANOVAS for comparisons among 23 °C, warm or cold ETZs.

ATP data: Data for CT_Max_ of *A. pisum* were non-normal, and transformation did not normalize the residuals, nonparametric ANOVAs (Kruskal–Wallis) were then used and followed by post-hoc nonparametric pairwise comparisons with Wilcoxon tests. ATP data sets from voluntary exposure to ETZs were analyzed following the same protocol as described previously for in crosses analysis of ETZ experiment. Longevity was analyzed using a two-way ANOVA with fungal load and thermal condition (critical temperature and behavior) as factors. Analyses were performed in the R programming environment (v. 3.4.3., CRAN project)^30^ and JMP-Pro version 15 (SAS Institute 2020).

## Results

### Critical thermal tolerance

**Figure 1:**
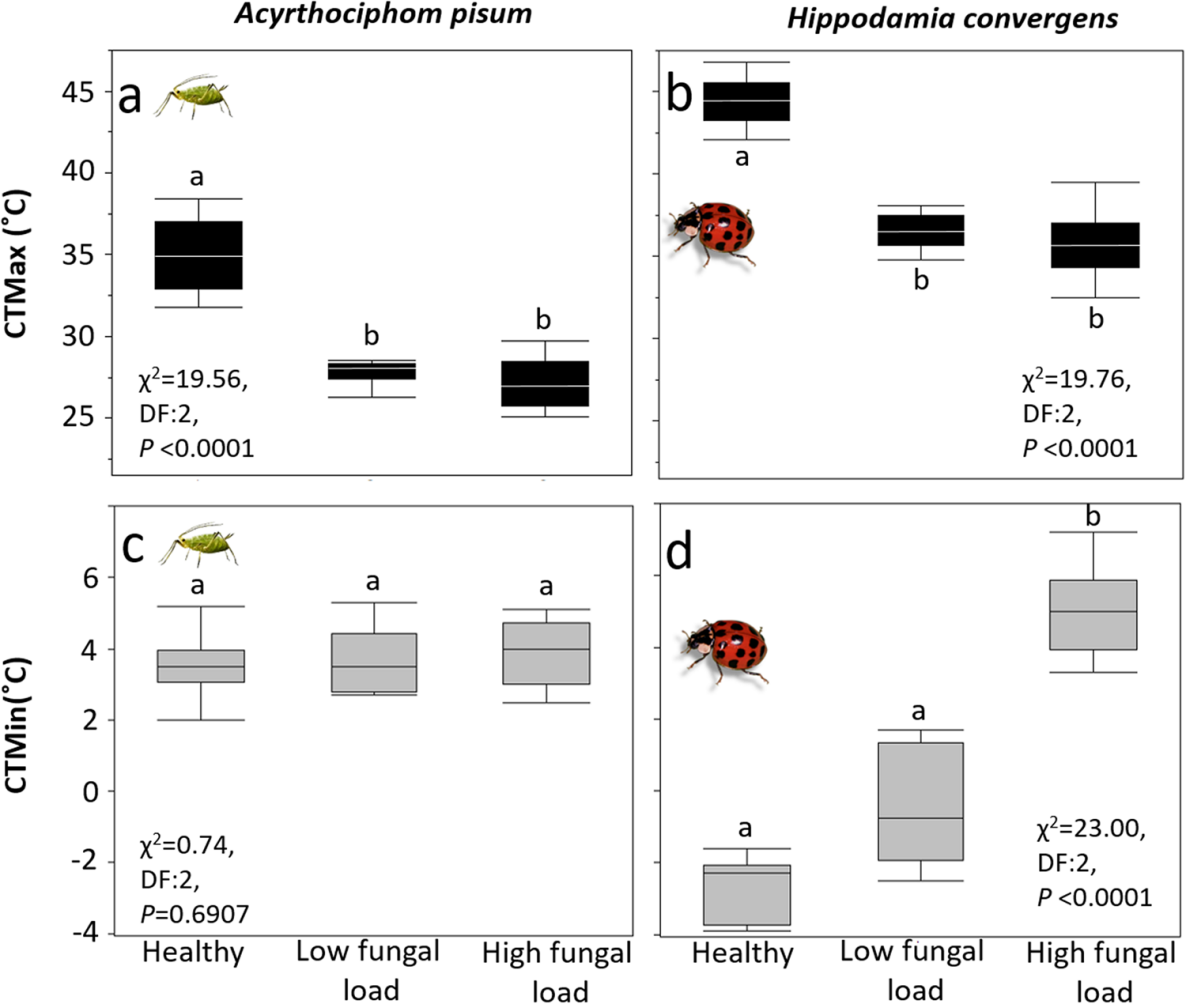
Maximum (CT_Max_) and minimum (CT_Min_) thermal tolerances for healthy and infected aphid and predator species. Low spore load = 1.4 × 10^6^ spores ha^−1^, high spore load = 1.4 × 10^12^ spores ha^−1^. CT_Max_ of (**a**) aphids (*A. pisum*) and (**b**) predator beetles (*H. convergens*). CT_Min_ of (**c**) aphids (*A. pisum*) and (**d**) predator beetles (*H. convergens*). Box plots display median line, interquartile range (IQR) boxes, 1.5 × IQR (*n* = 10 individuals per treatment). Black boxes: CT_Max_; Gray boxes: CT_Min_. Significance was determined by non-parametric ANOVA test, followed by multiple comparisons (*α* = 0.05) (*n* = 10).

Fungal infection reduced heat tolerance of *A. pisum* and *H. convergens* by an average 7 °C and 4 °C, respectively, regardless of the pathogen load, compared to healthy individuals (Fig. 1a,d). Regarding cold tolerance, the pathogen effect was species-specific, with infected aphids having comparable levels of cold tolerance relative to healthy ones, but predator beetles’ ability to withstand cold was reduced according to pathogen load (Fig. 1).

### Impacts of infection on voluntary exposure of aphids and predator beetles to extreme thermal zones

**Figure 2:**
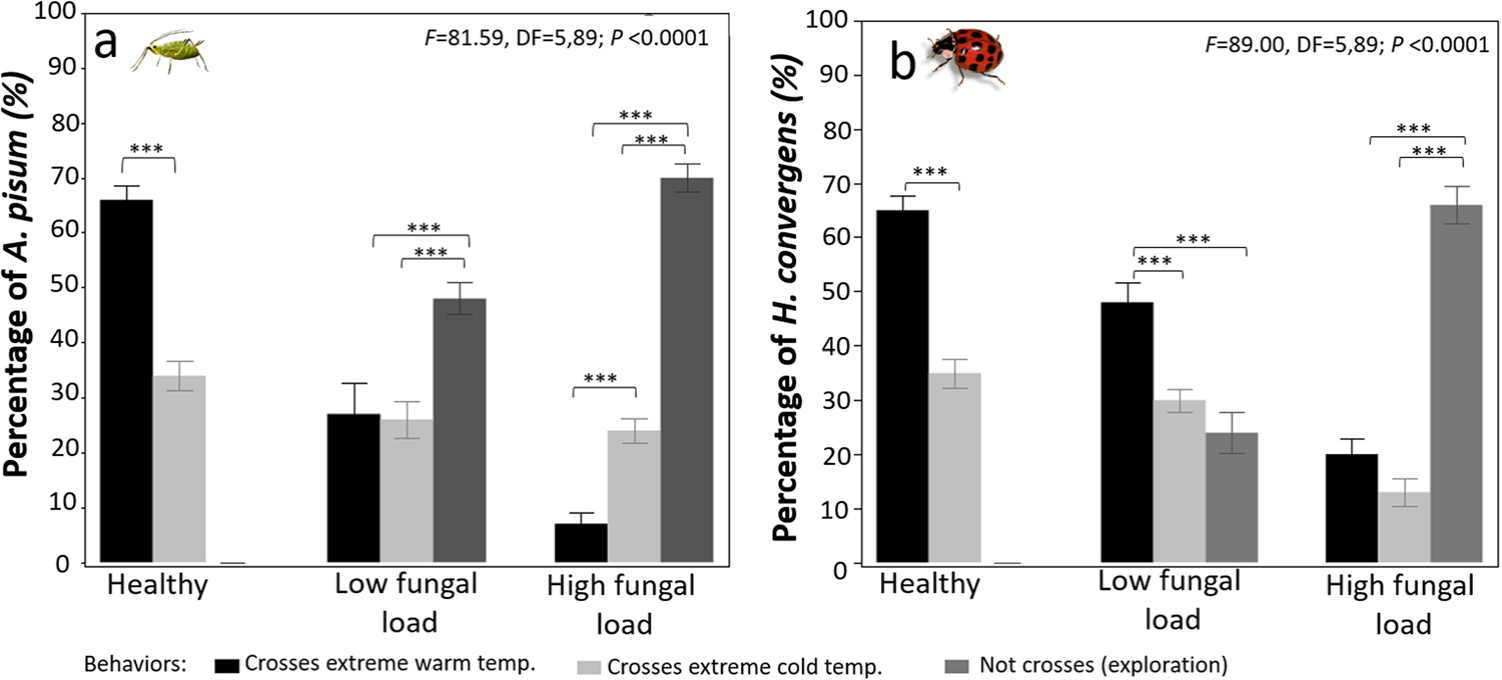
Thermal boldness of healthy and infected aphid and predator beetle species, measured as the number of individuals in a sample that voluntarily crossed through extreme thermal zones (ETZs, cold or warm). Infections: Low fungal load = 1.4 × 10^6^ spores ha^−1^, high fungal load = 1.4 × 10^12^ spores ha^−1^. (**a**) Aphids (*A. pisum*). (**b**) Predator beetles (*H. convergens*). Warm and cold ETZs for *A. pisum* were set at 48 °C and − 4 °C, whereas for *H. convergens* thermal barriers were set at 56 °C and − 12 °C. Two–way ANOVA. Bars represent mean ± SE, ***P < 0.0001 differences within treatments (replicates *n* = 10, 10 individuals per replicate, 100 individuals in total per treatment).

Healthy aphids and predator beetles were very bold and crossed ETZs, but in both species crosses through the warm ETZ were more frequent (> 60% of individuals in samples) than crosses through the cold ETZ (< 36%; Fig. 2). However, thermal boldness of both species was reduced by fungal infections in a dose-dependent manner. For instance, almost 50% of aphids infected with low fungal load opted not to cross ETZs and stayed inside the home bottle, and the remaining 50% crossed the warm (27%) or the cold (24%) ETZs in a similar fashion. When infected with the high fungal load, more aphids did not cross ETZs (70%), and less crossed through the warm ETZ (8%), while crosses through the cold ETZ remained comparable to those at the low fungal load. Similarly, 47% of infected beetles with a low fungal load crossed through the warm ETZ, 30% crossed through the cold ETZ, and 21% did not perform any cross. Most beetles infected with the high fungal dose did not cross any ETZ (65.5%), whereas 22.6% crossed the warm ETZ and only 12% crossed through the cold ETZ.

### Energetic costs associated with fungal infection of aphid and predator beetles under critical thermal limits and voluntary exposure to ET

**Figure 3:**
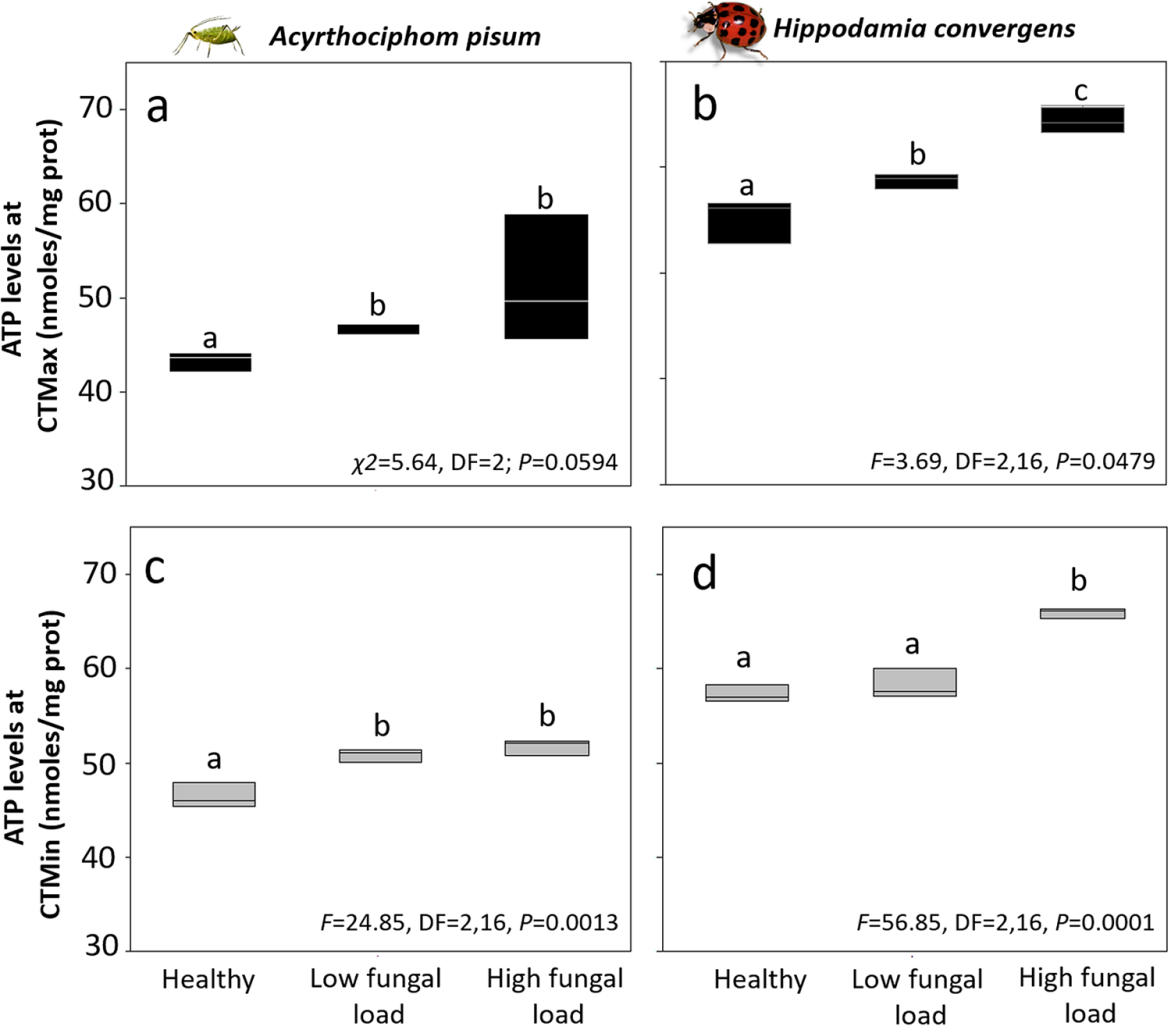
ATP levels of healthy and infected aphid and predator beetle species at upper and lower thermal tolerance (CT_Max_ or CT_Min_). Infections: Low spore load = 1.4 × 10^6^ spores ha^−1^, high spore load = 1.4 × 10^12^ spores ha^−1^**.** Box plots display median line, interquartile range (IQR) boxes, 1.5 × IQR (*n* = 3 individuals per treatment). Black boxes: CT_Max_; gray boxes: CT_Min_. Significance was determined by ANOVAs and non-parametric ANOVAs (Kruskal–Wallis) followed by *post-hoc* tests, letters represent differences within treatments (*α* = 0.05).

Although ATP levels of infected aphids at their CT_Max_ nominally increased with fungal load relative to healthy individuals, these differences were not statistically significant (Fig. 3a). Nevertheless, ATP levels of beetles exposed to their CT_Max_ significantly increased with fungal load, relative to healthy beetles (Fig. 3b). ATP levels at the CT_Min_ of aphids and predator beetles followed a similar trend, with ATP levels highest in infected individuals with the high fungal load, intermediate in infected aphids with low fungal load, and lowest in healthy ones (Fig. 3c,d).

**Figure 4:**
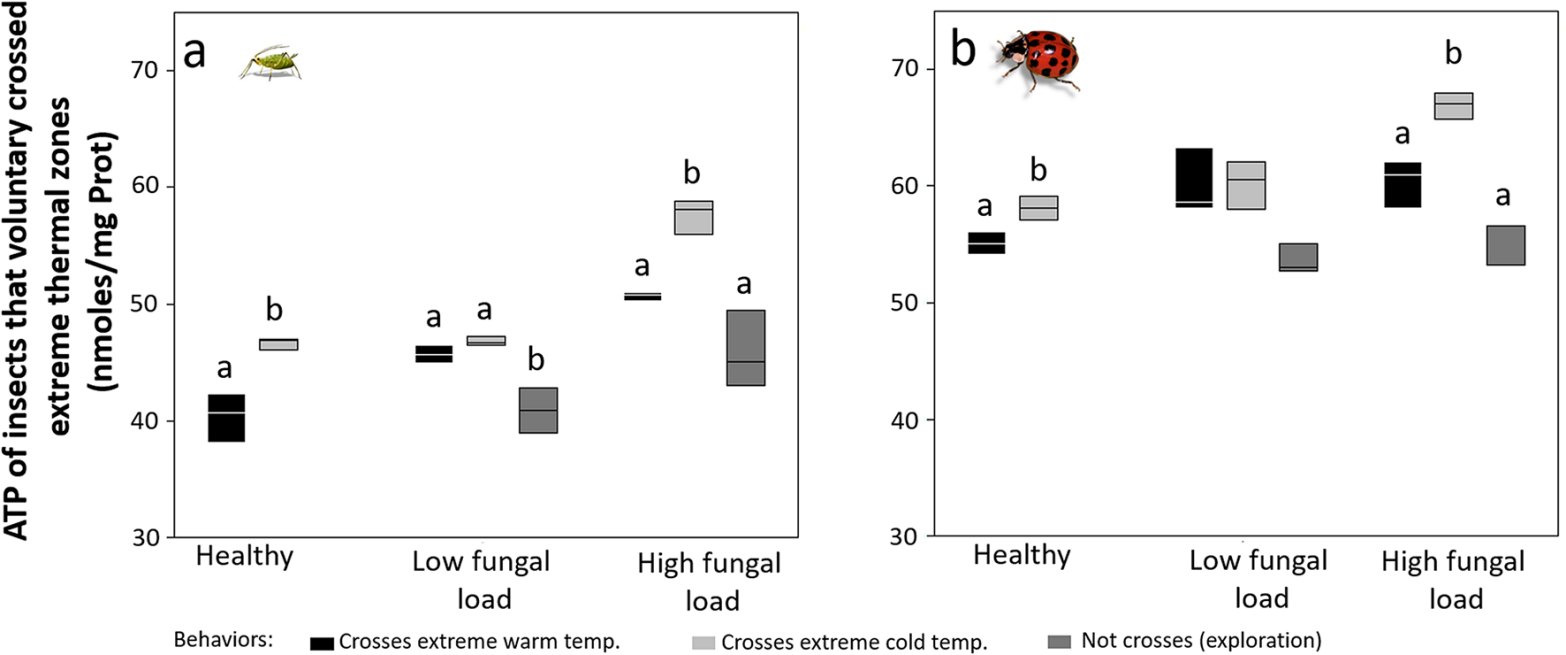
ATP levels of healthy and infected aphids (**a**) and predator beetles (**b**) that voluntarily crossed (or not) through extreme thermal zones (ETZ cold or warm). *A. pisum F* = 29.13 DF = 7,16, *P* < 0.001*. H. convergens*: *F* = 16.77, DF = 7,16; *P* < 0.0001 Infections: Low fungal load = 1.4 × 10^6^ spores ha^-1^, high fungal load = 1.4 × 10^12^ spores ha^-1^**.** Box plots display median line, interquartile range (IQR) boxes, 1.5 × IQR (*n* = 3 individuals per treatment). Significant difference was determined by Two-way ANOVA, followed by t-test for control treatment and multiple comparisons (*α* = 0.05) within low and high fungal load treatments. Letters represent differences within treatments. Black boxes: VT_Max_; dark gray boxes: no crosses (exploration); light gray: VT_Min_.

ATP levels of healthy aphids and predator beetles were significantly higher in individuals that crossed cold ETZs (aphid: *t* = 5.51, DF = 3.49, *P* = 0.0039; beetle: *χ*^*2*^ = 3.85, DF = 1, *P* = 0.0495) (Fig. 4). In infected aphids with low fungal loads, the highest ATP levels were found in individuals that crossed ETZs, but those levels did not significantly differ between cold and warm ETZs. Infected aphids that did not cross ETZs or remained at 23 °C showed the lowest ATP levels (*F* = 12.60, DF = 2,6, *P* = 0.0111). ATP levels in infected aphids with high fungal load followed the same trend (*F* = 24.27, DF = 2,6, *P* = 0.0013).

ATP levels did not significantly differ in infected beetles with low fungal doses (*χ*^*2*^ = 5.42, DF = 2; *P* = 0.0665). However, we found significant differences in ATP levels of beetles infected with high fungal loads. ATP levels were significantly higher in infected beetles that crossed cold ETZ, followed by ATP levels of beetles crossing the warm ETZ. Infected beetles that remained at 23 °C showed the lowest ATP levels (*χ*^*2*^ = 5.42, DF = 2, *P* = 0.0665).

### Effects of fungal infection and thermal conditions (critical thermal limits and voluntary exposure to ETZs) on longevity of aphids and predator beetles

**Figure 5:**
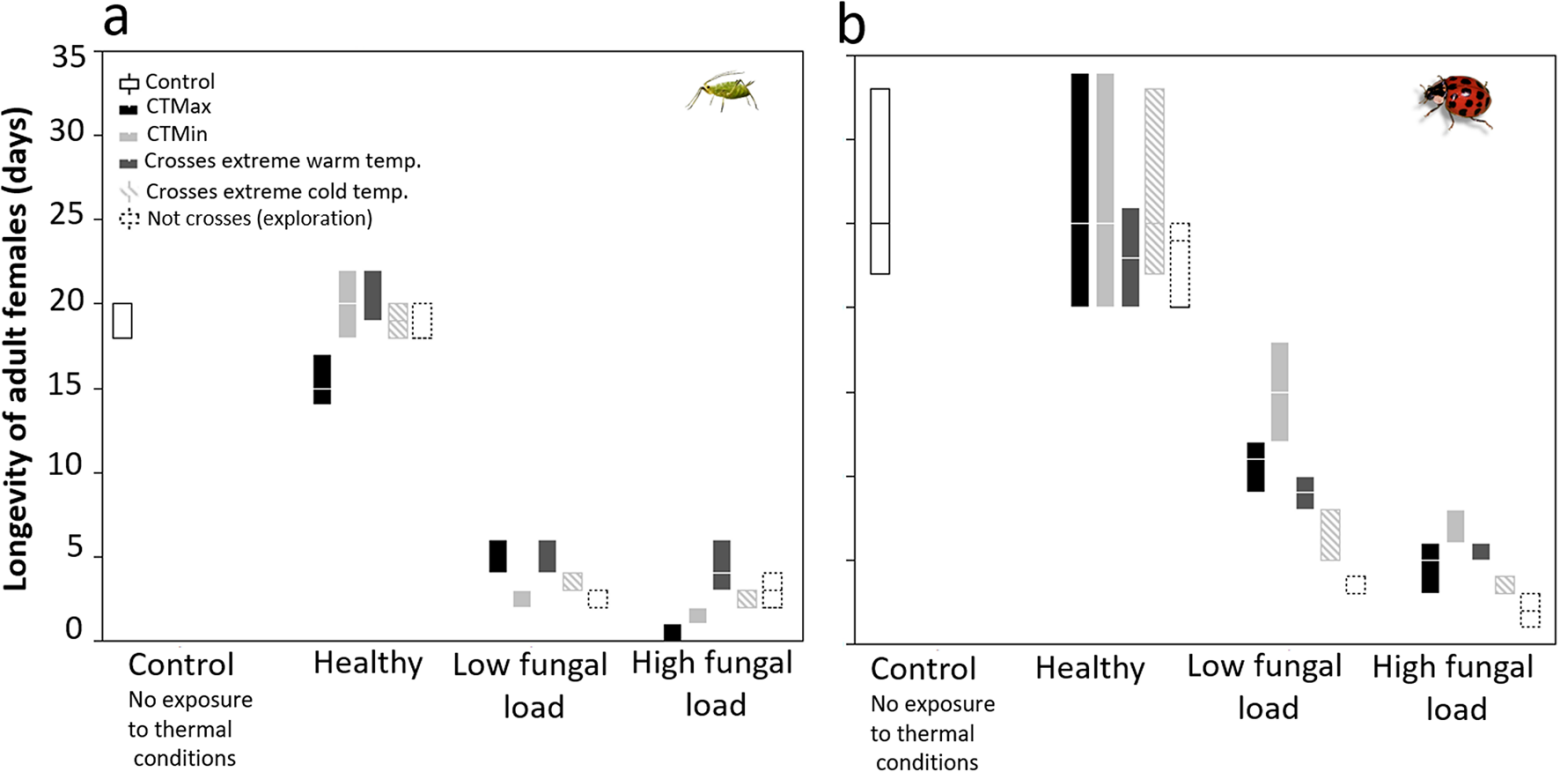
Longevity of adult healthy and infected aphids (**a**) and predator beetles (**b**) that experienced thermal conditions: control, healthy and infected individuals exposed to CT_Max_, CT_Min_, crosses to warm or cold ETZ, and exploration but no ETZ crosses. Healthy, low fungal load = 1.4 × 10^6^ spores ha^−1^, high fungal load = 1.4 × 10^12^ spores ha^−1^**.** Box plots display median line, interquartile range (IQR) boxes, 1.5 × IQR, *n* = 3 individuals per treatment.

The longevity of aphids was significantly reduced by fungal infection, thermal exposure (CT_Max_, CT_Min_ and ET), and the factor interaction by an average of 13.5 days (*F* = 152.11, DF = 17,36, *P* < 0.0001; *F* = 1253.64, DF = 2, *P* < 0.0001; *F* = 7.44, DF = 5, *P* < 0.0001; interactions infection*thermal response *F* = 4.15, DF = 10, *P* = 0.0007, PCR). Beetle longevity followed a similar trend, with both factors reducing longevity by average of 11.3 days (*F* = 31.17, DF = 17,90, *P* < 0.0001, *F* = 253.15, DF = 17,90, *P* < 0.0001; *F* = 2.40, DF = 5, *P* = 0.431; factor combination *F* = 4.15, DF = 10, *P* = 0.3192; Fig. 5, Supplementary Table 1).

## Discussion

The results of our field experiments demonstrate that fungal infection reduced the heat tolerance limits of aphids and predator beetles asymmetrically, while cold tolerance was only reduced in *H. convergens* (Fig. 1). Infection reduced voluntary exposure to ET in both aphid and predator (Fig. 2). Our results indicate that ATP levels are high in adult insects infected with high fungal loads and exposure to cold ET (Figs. 3, 4). Survival was significantly reduced with infection and thermal conditions (Fig. 5). Fungal infections in insects are expected to be compensated by behavioral fever^11^; however, this is the first time, to our knowledge, that infection shifts behavioral response for both predator and prey in ways that reduce exposure to heat.

The maximum heat tolerance was reduced by fungal infection, observed as narrowed limits with increasing fungal load in both aphids and predator beetles, suggesting that *B. bassiana* may have induced the opposite of host behavioral fevering. Fungal infection may block ion transfer in cells, challenging the homeostasis of insects^31,32^. Although behaviors that elevate body temperature are expected in infected insects^6^, our results indicated that infection altered boldness to cross ET. Reduced heat tolerance was confirmed in behavioral experiments as the majority of infected aphids and beetles were not able to cross either warm or cold ETZ, while the majority of healthy insects crossed either ETZ (Figs. 1, 2). This suggests that fungus might manipulate host physiology and behavior in ways that favor the fungal virulence^33^ since its thermal thresholds are narrower than any of the insect hosts^12,13,34^. However, infection is costly, and insects redirect energy to immune function to battle it, leaving less energy available to face thermal challenges.

The energetic cost of infection for both insect species at minimum thermal limits significantly increased with fungal load (Figs. 3, 4). This suggests that both factors, thermal condition and infection, may disrupt metabolic pathways resulting in increased ATP synthesis. ATP levels can increase following cold exposure in aphids^35^ and other insects, including flies^36^. Changes in ATP levels might be explained by a mismatch among metabolic pathways, since many cellular functions are ATP-dependent. For example, ion pumps, proteolysis, synthesis of substances used to prevent cell damage from cold (i.e., glycerol, trehalose, or proline)^37^, and expression of heat shock protein genes require ATP synthesis. Our results suggest that fungal infection and short exposure to critical limits or ETZ did not critically disrupt metabolic pathways since aphids and beetles were able to synthesize ATP, but might have long-lasting impacts on other parameters such as longevity and fitness. Further ecophysiological studies are needed to identify the mechanisms underlying the ATP synthesis under infection and ET.

Our field experiments indicate that exposure to the maximum thermal limit reduced longevity by about 3 days in aphids, and fungal infection significantly reduced longevity for both aphids and predator beetles (Fig. 5) exposed to any of the thermal conditions; 100% of insects used in the experiments that were infected died earlier than healthy individuals. Previous studies have suggested that the broad host range of *B. bassiana*^38^, which includes earthworms, beetles, parasitoids, and honey bees^39^, reported similar lethal and sublethal effects. For example, *B. bassiana* alters foraging and prey handling in the predator mite, *P. persimilis*^40^. The findings of this study suggest that longevity might be reduced by disruption of ATP synthesis in infected insects during exposure to thermal limits and ET.

We demonstrated that multiple stressors, such as ET and fungal infection, profoundly affect thermal tolerance in aphids and predator beetles, but in different ways, leading to an asymmetric response. Thermal stress and infection may alter predators’ foraging, handling time, and digestion^41^. Altogether, the results indicate the strong effects that infection has on thermal tolerance across trophic levels. This finding suggests that a fungus can modify behavioral plasticity with implications for thermoregulatory strategies in insects with cascading impacts on the strengths of predator–prey interactions and food webs. Additional studies are needed to understand the molecular and physiological mechanisms underlying reduced thermal boldness or voluntary exposure to ETZ in infected insects. This information has critical implications for understanding the physiological and behavioral mechanisms by which organisms respond to biotic and abiotic stressors. Overall, the findings suggest that we cannot expect to understand how an organism responds to the environment by studying the insect species alone, as pathogen infections can alter insect thermal physiology and behavior. This new dimension opens a wide array of research avenues with fundamental and applied implications to the management of insect species.

## Supplementary Information

Supplementary Information.

## Data Availability

The source data underlying Figs. 1, 2, 3, 4 and 5 have been deposited in TERN data repository, identifier b9c3747a-f2ba-4c50-9349-2ddf0ab4d09a [https://geonetwork.tern.org.au/geonetwork/srv/eng/catalog.search#/metadata/b9c3747a-f2ba-4c50-9349-2ddf0ab4d09a].

## References

[1] Cory JS, Ericsson JD (2009). Fungal entomopathogens in a tritrophic context. The Ecology of Fungal Entomopathogens.

[2] Richards-Zawacki CL (2010). Thermoregulatory behaviour affects prevalence of chytrid fungal infection in a wild population of Panamanian golden frogs. Proc. R. Soc. B: Biol. Sci..

[3] Caillon R (2014). Warming decreases thermal heterogeneity of leaf surfaces: Implications for behavioural thermoregulation by arthropods. Funct. Ecol..

[4] Potter KA, Arthur Woods H, Pincebourde S (2013). Microclimatic challenges in global change biology. Glob. Change Biol..

[5] Paudel S (2020). Asymmetric responses to climate change: Temperature differentially alters herbivore salivary elicitor and host plant responses to herbivory. J. Chem. Ecol..

[6] Elliot SL, Blanford S (2002). Thomas MB (2002) Host–pathogen interactions in a varying environment: Temperature, behavioural fever and fitness. Proc. R. Soc. Lond. Ser. B. Biol. Sci..

[7] Adamo S (1998). The specificity of behavioral fever in the cricket *Acheta domesticus*. J. Parasitol..

[8] Louis C, Jourdan M, Cabanac M (1986). Behavioral fever and therapy in a rickettsia-infected Orthoptera. Am. J. Physiol.-Regul. Integr. Comp. Physiol..

[9] Blanford S, Read AF, Thomas MB (2009). Thermal behaviour of *Anopheles stephensi* in response to infection with malaria and fungal entomopathogens. Malar. J..

[10] Williams CM (2016). Biological impacts of thermal extremes: Mechanisms and costs of functional responses matter. Integr. Comp. Biol..

[11] Thomas MB, Blanford S (2003). Thermal biology in insect–parasite interactions. Trends Ecol. Evol..

[12] Membang G (2021). Thermal response and horizontal transmission of cameroonian isolates of the entomopathogenic fungi *Beauveria bassiana* and Metarhizium anisopliae—Candidates for microbial controls of the banana root borer Cosmopolites sordidus. Fungal Ecol..

[13] Fargues J (1997). Effect of temperature on vegetative growth of *Beauveria bassiana* isolates from different origins. Mycologia.

[14] Lamb R (1992). Developmental rate of *Acyrthosiphon pisum* (Homoptera: Aphididae) at low temperatures: Implications for estimating rate parameters for insects. Environ. Entomol..

[15] Katsarou I (2005). Effect of temperature on development, growth and feeding of *Coccinella septempunctata* and *Hippodamia convergens* reared on the tobacco aphid, *Myzus persicae* nicotianae. Biocontrol.

[16] McCue MD, De Los Santos R (2013). Upper thermal limits of insects are not the result of insufficient oxygen delivery. Physiol. Biochem. Zool..

[17] Portner H (2002). Climate change and temperature dependent biogeography: Systemic to molecular hierarchies of thermal tolerance in animals. Comp. Biochem. Physiol. A.

[18] Zhang H (2018). Life-history traits buffer against heat wave effects on predator–prey dynamics in zooplankton. Glob. Change Biol..

[19] Smolinský R, Gvoždík L (2014). Effect of temperature extremes on the spatial dynamics of predator–prey interactions: A case study with dragonfly nymphs and newt larvae. J. Therm. Biol.

[20] Grigaltchik VS, Ward AJ, Seebacher F (2012). Thermal acclimation of interactions: Differential responses to temperature change alter predator–prey relationship. Proc. R. Soc. B Biol. Sci..

[21] Roy HE (2006). Bizarre interactions and endgames: Entomopathogenic fungi and their arthropod hosts. Annu. Rev. Entomol..

[22] Hochachka PW, Somero GN (2002). Biochemical Adaptation: Mechanism and Process in Physiological Evolution.

[23] MacMillan HA, Sinclair BJ (2011). The role of the gut in insect chilling injury: Cold-induced disruption of osmoregulation in the fall field cricket, *Gryllus pennsylvanicus*. J. Exp. Biol..

[24] Navas CA, Agudelo-Cantero GA, Loeschcke V (2012). Thermal boldness: Volunteer exploration of extreme temperatures in *Drosophila melanogaster*. bioRxiv.

[25] Ribeiro PL, Camacho A, Navas CA (2012). Considerations for assessing maximum critical temperatures in small ectothermic animals: Insights from leaf-cutting ants. PLoS ONE.

[26] Churchill TA, Storey KB (1989). Metabolic consequences of rapid cycles of temperature change for freeze-avoiding vs freeze-tolerant insects. J. Insect Physiol..

[27] Barbarin AM (2012). A preliminary evaluation of the potential of *Beauveria bassiana* for bed bug control. J. Invertebr. Pathol..

[28] Wraight S, Ramos M (2002). Application parameters affecting field efficacy of *Beauveria bassiana* foliar treatments against Colorado potato beetle *Leptinotarsa decemlineata*. Biol. Control.

[29] Castrillo L, Vandenberg J, Wraight S (2003). Strain-specific detection of introduced *Beauveria bassiana* in agricultural fields by use of sequence-characterized amplified region markers. J. Invertebr. Pathol..

[30] Team, R.C., *R: A Language and Environment for Statistical Computing* (2013).

[31] Clarkson JM, Charnley AK (1996). New insights into the mechanisms of fungal pathogenesis in insects. Trends Microbiol..

[32] Altinok HH, Altinok MA, Koca AS (2019). Modes of action of entomopathogenic fungi. Curr. Trends Nat. Sci.

[33] Hughes D (2016). From so simple a beginning: The evolution of behavioral manipulation by fungi. Adv. Genet..

[34] Stoks R (2017). Daily temperature variation and extreme high temperatures drive performance and biotic interactions in a warming world. Curr. Opin. Insect Sci..

[35] Pullin, A. & Bale, J. Cause and effects of pre-freeze mortality in aphids. *Cryo-Letters* (1988).

[36] Colinet H (2011). Disruption of ATP homeostasis during chronic cold stress and recovery in the chill susceptible beetle (*Alphitobius diaperinus*). Comp. Biochem. Physiol. A: Mol. Integr. Physiol..

[37] Toxopeus J, Sinclair BJ (2018). Mechanisms underlying insect freeze tolerance. Biol. Rev..

[38] James R, Lighthart B (1994). Susceptibility of the convergent lady beetle (Coleoptera: Coccinellidae) to four entomogenous fungi. Environ. Entomol..

[39] Zimmermann G (2007). Review on safety of the entomopathogenic fungi *Beauveria bassiana* and *Beauveria brongniartii*. Biocontrol Sci. Technol..

[40] Seiedy M (2012). Functional response of *Phytoseiulus persimilis* (Acari: Phytoseiidae) on untreated and *Beauveria bassiana*-treated adults of *Tetranychus urticae* (Acari: Tetranychidae). J. Insect Behav..

[41] Sentis A, Hemptinne J-L, Brodeur J (2012). Using functional response modeling to investigate the effect of temperature on predator feeding rate and energetic efficiency. Oecologia.

