# Supplementary material for: Fungal infections lead to shifts in thermal tolerance and voluntary exposure to extreme temperatures in both prey and predator insects
Source: Sci Rep. 2021 Nov 5;11:21710. doi: 10.1038/s41598-021-00248-z (PMC8571377; doi:10.1038/s41598-021-00248-z)
Supplement: Supplementary file 1 — Supplementary Information. [file 41598_2021_248_MOESM1_ESM.pdf]

Fungal infections lead to shifts in thermal tolerance and voluntary exposure to extreme temperatures in both prey and predator insects

Supplementary information 1

Mitzy F. Porras[1*], Gustavo A. Agudelo-Cantero[2,3], M Geovanni Santiago-Martinez[4], Carlos A. Navas[2], Volker Loeschcke[3], Jesper Givskov Sørensen[3], Edwin G. Rajotte[1]

[1] Department of Entomology, The Pennsylvania State University, 501 ASI Bldg. University Park, PA 16802, USA

[2] Department of Physiology, Institute of Biosciences, University of São Paulo, Rua do Matão 101, Tv 14, 05508-090 São Paulo, Brazil

[3] Department of Biology – Genetics, Ecology and Evolution, Aarhus University, Ny Munkegade 116, DK-8000 Aarhus C, Denmark

[4] Department of Biochemistry, The Pennsylvania State University, 308B Althouse Lab. University Park, PA 16802, USA

*Corresponding author: Mitzy Porras, E-mail: mitzy.porras@gmail.com, +1 765 409 2795, Fax: 814 865-3048

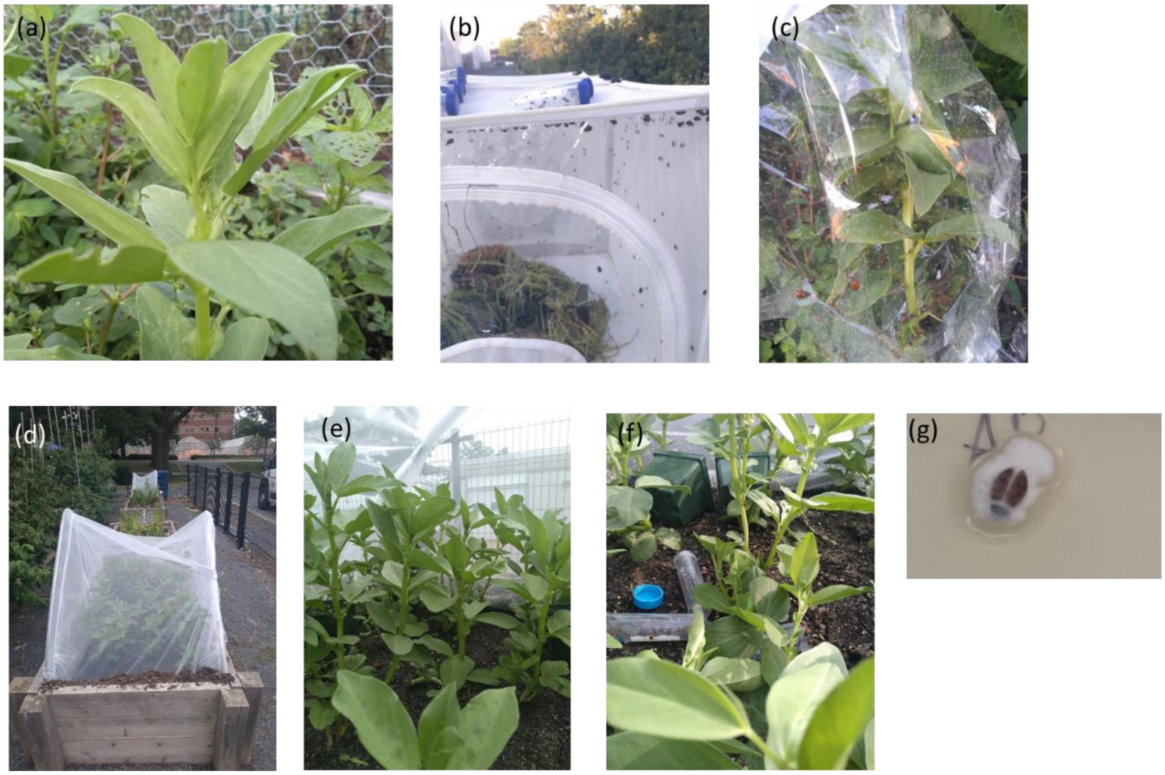

Figure S1. Experimental microcosms testing the effects fungal infections on the thermal tolerance and volunteer exposure to extreme temperatures of prey and predator insect species. Colonies and experiments under field conditions (a) *Acyrthociphom pisum* and (b-c) *Hippodamia convergens*. (d-f) Experimental microcosms to evaluate two fungal loads: low spore load= 1.4 x 10 ^{6}spores ha^{-1}, high spore load = 1.4 x 10^{12} spores ha^{-1}. (g) *Hippodamia convergens* adults infected with high fungal loads.

Table S1 Spores on experimental individuals of *Acyrtociphom pisum* and *Hippodamia convergens* subject thermal conditions (CT_{Max}, CT_{Min}, voluntary exposure to warm or cold ETZs (Mean ±s.e.)

**Treatment**			**Spores (Mean ±s.e.)**		
Acyrtocyphon pisum	**CT_{Max}**	**CT_{Min}**	**Exploration**	**Warm ETZ**	**Cold ETZ**
**Healthy**	0.16 ± 0.09	0.5 2± 0.15	0	0.2 ± 0.10	0.54 ±0.14
**Low fungal load**	6.37 ± 0.68	0.95 ± 0.27	10.7 ± 0.74	1.08 ± 0.25	8.04 ±0.86
**High fungal load**	11.33± 0.85	25.5 ± 2.01	23.25 ± 0.97	6.79 ± 0.95	16.5 ±0.98
Hippodamia convergens					
**Healthy**	0.125 ± 0.06	0.04 ± 0.06	0.04 ± 0.04	0.41 ± 0.28	0.04 ± 0.04
**Low fungal load**	7.33 ± 1.09	22.37 ± 1.28	13.75 ± 1.3	3.37 ± 0.87	13.75 ± 1.12
**High fungal load**	24.83 ± 1.45	23.41 ± 1.42	14.95 ± 1.78	12.58 ± 0.89	14.95 ± 0.91

Table S2. Parameter estimates of analysis of deviance (type II) tables of full-factorial models from experiments testing the effect fungal infections and exposure to critical and extreme temperatures on *A. pisum* (AIC=226.59)

**Term**	**Estimate**	**Std Error**	**L-R χ^2**	**Prob> χ^2**	**Lower CL**	**Upper CL**
Intercept	1.6783181	0.1812863	47.997587	<.0001*	1.3001056	2.0158612
Treatment[Healthy]	1.2474324	0.1837697	57.752738	<.0001*	0.903057	1.6290838
Treatment[High load]	-0.849709	0.2748498	12.158488	0.0005*	-1.446586	-0.351222
Exp[control no therm]	0.4719357	0.8862827	0.2815829	0.5957	-1.301294	2.2213609
Exp[Ctmax]	-0.621031	0.3897594	3.319932	0.0684	-1.631974	0.042479
Exp[Ctmin]	-0.182522	0.2626325	0.4881446	0.4848	-0.712676	0.3279798
Exp[vtmax]	0.3670305	0.2199004	2.859498	0.0908	-0.057837	0.8099065
Exp[Vtmin]	0.0186885	0.121194	0.0236617	0.8777	-0.226755	0.2492391
Exp[control no therm]*Treatment[Healthy]	-0.435855	0.8784206	0.2445187	0.6210	-2.16945	1.3235993
Exp[control no therm]*Treatment[High load]	0.8466504	1.3705212	0.3777615	0.5388	-1.931149	3.6352916
Exp[Ctmax]*Treatment[Healthy]	0.4253094	0.403089	1.3193899	0.2507	-0.271528	1.4509115

Term	Estimate	Std Error	L-R χ^2	Prob> χ^2	Lower CL	Upper CL
Exp[Ctmax]*Treatment[High load]	-1.306191	0.7282436	4.7250835	0.0297*	-3.274048	-0.113369
Exp[Ctmin]*Treatment[Healthy]	0.2525042	0.2780105	0.8337313	0.3612	-0.288591	0.8107999
Exp[Ctmin]*Treatment[High load]	-0.135261	0.4244667	0.1022222	0.7492	-1.005281	0.6933132
Exp[vtmax]*Treatment[Healthy]	-0.297049	0.2380549	1.5846084	0.2081	-0.773954	0.1639418
Exp[vtmax]*Treatment[High load]	0.2706972	0.3442771	0.6324496	0.4265	-0.390072	0.9775987
Exp[Vtmin]*Treatment[Healthy]	0	.	.	.	.	.
Exp[Vtmin]*Treatment[High load]	0	.	.	.	.	.

Table S3. Parameter estimates of analysis of deviance (type II) tables of full-factorial models from experiments testing the effect fungal infections and exposure to critical and extreme temperatures on *H. convergens* (AICc=268.44).

**Term**	**Estimate**	**Std Error**	**L-R χ^2**	**Prob> χ^2**	**Lower CL**	**Upper CL**
Intercept	2.1227029	0.1427327	105.80056	<.0001*	1.8297187	2.3918986
Treatment[Healthy]	1.1072119	0.1447328	69.552011	<.0001*	0.8326525	1.4030131
Treatment[High load]	-0.87692	0.2169883	21.507157	<.0001*	-1.338942	-0.479615
Exp[control no therm]	-0.614136	0.6546677	0.8981856	0.3433	-1.932339	0.6443899
Exp[Ctmax]	0.2700986	0.1782733	2.3129418	0.1283	-0.077972	0.6242407
Exp[Ctmin]	0.5026324	0.168515	9.2306397	0.0024*	0.1769253	0.8403538
Exp[vtmax]	0.2330704	0.1761357	1.771915	0.1831	-0.109584	0.5838873
Exp[Vtmin]	0.0534996	0.1026458	0.2679036	0.6047	-0.153211	0.2497109
Exp[control no therm]*Treatment[Healthy]	0.6676359	0.6473095	1.0896016	0.2966	-0.57483	1.9730426
Exp[control no therm]*Treatment[High load]	-0.321543	0.9668145	0.1124888	0.7373	-2.331409	1.4939472

Term	Estimate	Std Error	L-R χ^2	Prob> χ^2	Lower CL	Upper CL
Exp[Ctmax]*Treatment[Healthy]	-0.229178	0.1948902	1.3915994	0.2381	-0.615347	0.1513134
Exp[Ctmax]*Treatment[High load]	0.0245631	0.2893245	0.0072149	0.9323	-0.541167	0.6042516
Exp[Ctmin]*Treatment[Healthy]	-0.461712	0.1860058	6.331376	0.0119*	-0.832673	-0.101268
Exp[Ctmin]*Treatment[High load]	0.1487042	0.2705213	0.3064098	0.5799	-0.371623	0.6983828
Exp[vtmax]*Treatment[Healthy]	-0.327491	0.1950384	2.8559736	0.0910	-0.714817	0.0520809
Exp[vtmax]*Treatment[High load]	0.2557473	0.2809862	0.8412902	0.3590	-0.288407	0.8229832
Exp[Vtmin]*Treatment[Healthy]	0	.	.	.	.	.
Exp[Vtmin]*Treatment[High load]	0	.	.	.	.	.
